# Biological responses in pesticide exposed lizards (*Podarcis siculus*)

**DOI:** 10.1007/s10646-021-02440-3

**Published:** 2021-06-26

**Authors:** Giulia Simbula, Ginevra Moltedo, Barbara Catalano, Giacomo Martuccio, Claudia Sebbio, Fulvio Onorati, Luca Stellati, Alessandra Maria Bissattini, Leonardo Vignoli

**Affiliations:** 1 Dipartimento di Scienze, Università Roma Tre, Viale G. Marconi 446, 00146 Rome, Italy; 2 Istituto Superiore per la Ricerca e la Protezione Ambientale - ISPRA, Via di Castel Romano, 100, Rome, Italy

**Keywords:** Pesticides, Field Study, Reptiles, Multi-biomarker approaches

## Abstract

The release of contaminants as herbicides, fungicides and insecticides into the environment has been listed as one of the six major contributors to the global decline of reptiles. Although reptiles may face severe risk from contaminants due to their ecology and physiology, they are currently less studied than other vertebrate groups. In the present work, we investigated if and how different types of field treatment (conventional and organic) affected the health status of Italian wall lizard (*Podarcis siculus*) individuals in central Italy. We chose a multi-biomarker approach that evaluated the biological responses of lizards to the treatment by means of AChE activity in the nervous system, biotransformation enzymes activities and oxidative stress in the liver, micronuclei frequency measured in the erythrocytes, and rate of intestinal parasitic infection. Our findings showed evidence of effects of treatment in conventional areas and between sexes with significant oxidative stress due to hydroxyl radicals, that caused DNA damage. No difference of intestinal parasite infections was found among treatments. *Podarcis siculus* seems to be a good bioindicator in ecotoxicological studies and potentially in risk assessment of pesticides, although further analyses in laboratory and in the field are needed to achieve more accurate quantification of specific pesticide effects in relation to known exposure history and to understand if other mechanisms were involved in the toxicity and detoxification process of pesticides for this species.

## Introduction

The intensive release of pesticides as insecticides, herbicides and fungicides in the modern agriculture has been listed as one of the major contributor to the global decline of reptiles (Gibbons et al. 2000; Todd et al. 2010; Mingo et al. 2017). Some reptiles species, especially lacertid lizards, are abundantly widespread in agricultural areas (Wisler et al. 2008; Freedberg et al. 2011, Bicho et al. 2013) where they often occur at high densities and biomass levels (Pough 1980). Reptiles, as non-target organisms for pesticides, can be directly and indirectly exposed to these contaminants through various routes, including (a) inhalation (Doya et al. 2020), (b) absorption from the skin (Weir et al. 2016; Mestre et al. 2019), (c) ingestion of contaminated food or soil (Mingo et al. 2017; Verderame and Scudiero 2019), (d) maternal transfer to eggs/ young (Weir et al. 2010, 2015), (e) absorption by eggs of contaminants from surrounding environments (Schaumburg et al. 2016), and (f) through alterations in the availability of food or microhabitats (i.e. plant cover) (Marco et al. 2004). The significant role of reptiles in ecosystems has been widely established and their exposure to these chemicals is proved and widespread, with consequences ranging from hormonal changes and enzymatic responses (i.e., oxidative stress, neurotoxic implications and immunosuppression) to physiological reactions like impairments in fertility, development and locomotor performance (DuRant et al. 2007; Amaral et al. 2012a, b, c; Bicho et al. 2013; Carpenter et al. 2016; Schaumburg et al. 2016). However, the actual effects of pesticides on reptiles, the existence of synergistic effects, and the biological factors involved in the defence response are still unclear and scarcely investigated, and they have been mainly inferred indirectly from surrogate species (birds, mammals and fish) (De Falco et al. 2007; Sparling et al. 2010; Weir et al. 2010). Reptiles remain the only vertebrates (along with amphibians) not directly considered in the environmental risk assessment (ERA) of pesticides. Only recently, the European Food Safety Authority highlighted the need for timely actions to fill such considerable gap of knowledge (EFSA et al. 2018).

Pesticides are known to primarily act on both target and non-target organisms through biochemical and molecular processes (Amiard-Triquet et al. 2012). However, they can propagate through higher levels of biological organization (cells, tissues and organs) producing cascading consequences on organisms and populations (Fossi and Leonzio 1993). Given this complex scenario characterized by a suite of possible effects involving different processes and scales, preliminary studies suggested to approach the analysis of pesticide impact on reptiles by combining the use of different cellular and molecular biomarkers in order to detect early-warning signals of contaminant exposure or effect, together with analysis at organism level such as body condition or intestinal parasitic infection (Livingstone 1993; Capriglione et al. 2011).

The main goal of the present study is to assess the effects of some pesticides exposure (thiophanate methyl TM, tebuconazole, lambda-cyhalothrin and deltamethrin) and other contaminants commonly used in hazelnut management activities on *Podarcis siculus* (Rafinesque-Schmaltz 1810) in Central Italy by means of multi biomarker approach. The set of biomarkers selected for this study involves acetylcholinesterase, glutathione-S- transferases activities, TOSC Assay and frequency of micronuclei.

Fungicides, such as tebuconazole, and pyrethroid insecticides, such as lambda-cyhalothrin and deltamethrin (and/or their metabolites) are known to inhibit the activity of acetylcholinesterase (AChE), to induce oxidative stress and to stimulate some detoxification processes in vertebrates, especially fishes (i.e., *Danio rerio*) (Altenhofen et al. 2017; Ullah et al. 2019). The fungicide TM, in general, has a lower toxicity compared with other common fungicides, however it and its derived products have been classified by U.S. EPA as probable human carcinogens (Capriglione et al. 2011). Even if data on the toxicity of TM are conflicting (Capriglione et al. 2011), it resulted to be genotoxic especially for humans and rats (Ben Amara et al. 2014). In this study, due the presence of mixed pesticides, including pyrethroid and ester carbamate, we hypothesized to find higher neurotoxic effect (i.e. inhibition of AChE) and alteration of the antioxidant system (measured by TOSC Assay towards peroxyl and hydroxyl radicals - TOSCA ROO^.^ TOSCA HO^.^) in lizards from conventional fields than in organic or control fields. We also expected activation of detoxification systems (i.e. GSTs) and cellular damages at DNA level (MN test). Moreover, we expected that pesticide exposure (i) by weakening the animals, increases the impact (i.e. abundance) of intestinal parasites of lizards from conventional fields; or (ii) directly affects the parasites, decreasing their prevalence within individuals of exposed sites. The Italian wall lizard was chosen as model species for its wide distribution in agricultural habitats (Bologna et al. 2000; Maura et al. 2011) due to its generalist habit (Corti et al. 2009) and dispersal ability (Vignoli et al. 2012). Moreover, the use of this lizard as a terrestrial bioindicator for ecotoxicological assessment of the impact of pollutants has been proposed and validated in different studies, focusing especially on endocrine dysfunction, impairments in fertility and reproduction success (De Falco et al. 2007; Sciarrillo et al. 2008; Marsili et al. 2009; Cardone 2015; Guerriero et al. 2018; Verderame and Scudiero 2019). Preliminary studies on effects of pesticides in similar species such as *P. muralis* (Laurenti 1768) and *P. bocagei* (Lopez-Seoane 1885), both in field and lab, suggested alterations in morphology, condition index, fertility, detoxicant and antioxidant systems, DNA (Amaral et al. 2012a; Capriglione et al. 2011; Cardone 2015; Mingo et al. 2016, 2017), although most of them are related to pesticides other than those investigated in this study (i.e. glyphosate, chlorpyrifos).

## Materials and methods

### Study area

Fieldwork was carried out in five localities in Viterbo province (Latium, central Italy; 507 m a.s.l.). This area has been extensively used for hazelnut management activities for more than 50 years and it is regularly being treated with synthetic pesticides and natural occurring pesticides as copper sulphate in order to control pests throughout the years (Carbone et al. 2004; Fabi and Varvaro 2010; Roversi 2016). The current and historical land and pesticide usage were obtained through consultation with the landowners. Study sites were all hazelnut cultivations and included (i) two conventional farming areas (T1, 42°21'26.20“N 12°16'11.55“E; T2, 42°20'43.72“N 12°24'5.80“E) where fungicides and insecticides have been applied routinely twice a years from May to July, (ii) two organic farming areas (B1, 42°21'45.53“N 12°15'36.86“E; B2, 42°26'24.70“N 12°18'46.30“E), where copper sulphate have been used once a year, and (iii) one control area (C, 42°19'38.7“N 12°02'52.3“E) pesticide free, with no history of chemical application for at least 10 years also in the neighbouring agricultural fields, surrounded by wild oak forest and sufficiently distant from conventional fields to discharge any effect of possible pollutants used in the surroundings (Table 1). In these environment, Italian wall lizards exploit the hazelnut roots, trunks, and low branches as well as the surroundings natural dry vegetation as the main habitat elements (e.g. for thermoregulation, foraging, as hiding place or even as deposition site). All the selected fields had similar geology, soil types and climate, and they were separated by at least 1-2 km, so that the interchange of individuals was considered unlikely in the short term.

**Table 1:** Applied chemicals and their application rates (field dose, kg-L/ha) in the sampling sites during the year 2018

Treatment	Area	ha	Chemical	Active ingredient	Kg-L/ha	Type
Conventional	T1	2	Ares 430sc®	Tebuconazole	0.20	Fungicide
			Sparviero®	Lambda-cyhalothrin	0.20	Insecticide
	T2	4	Ares 430sc®	Tebuconazole	0.50	Fungicide
			Copper 40%	Copper sulphate	3	Herbicide/bactericide/fungicide
			Sparviero®	Lambda-cyhalothrin	0.25	Insecticide
			Enovit®	Thiophanate-methyl	1.7	Fungicide
			Glorial 25ec®	Deltamethrin	0.5	Insecticide
Organic	B1	2.5	Copper	Copper sulphate	2.2	Herbicide/bactericide/fungicide
	B2	2	Copper	Copper sulphate	2.2	Herbicide/bactericide/fungicide
Control	C	3	–	–	–	

*ha* area surface in hectare

### Lizard sampling and lab design

Sampling activity took place from June to July 2018. Seven days from pesticide application (Table 1 “Appendix”), adult lizards were captured by noose or hand, sexed using sexual secondary characters (Arnold and Ovenden 2002), measured for body mass (BM) (Digital scale, DIPSE TP 2000, precision 0.1 g), for snout-vent length (SVL) with a calliper (precision 0.01 mm) and transported to the Italian Institute for Environmental Protection and Research (ISPRA) laboratories (Castel Romano, Latium, central Italy) where they were killed by cervical dislocation and dissected (Amaral et al. 2012a). Brain, liver, blood, and gastro-intestinal tract were removed, weighted, and used for the biomarker analyses. Brain and liver were frozen in liquid nitrogen and kept at −80 °C, while the digestive tract was preserved in 96 % ethanol. Blood samples were gathered from the caudal tail vein using a heparinised needle, fixed in Carnoy’s solution (methanol: acetic acid = 3:1) and stored at 4 °C.

### Studied biomarkers

The acetylcholinesterase (AChE) and glutathione-S-transferases (GSTs) activities are well established and suitable biomarkers for pesticides exposure in lacertid lizards (Mingo et al. 2017).

The AChE is an enzyme with a fast activity that catalyses the breakdown of acetylcholine and other choline esters that function as neurotransmitters (Quinn 1987). Since it is the primary target of inhibition by organophosphorus, carbamate and pyrethroid compounds (Tougu 2001), it has been commonly used to assess neurotoxic pesticide effects on organisms (Day and Scott 1990).

The GSTs, a family of phase II metabolic enzymes, catalyse the conjugation of the reduced form of glutathione (GSH) to xenobiotic substrates having electrophilic centres (nitrocompounds, organophosphates, organochlorines), making them more water-soluble facilitating their excretion. These enzymes exist in multiple forms and their activities have been also associated with resistance to the major classes of pesticides since they can be altered by a wide range of them (Domingues et al. 2010; Mingo et al. 2017).

Oxidative stress quantification and the frequency of micronuclei (MN) could be considered as widespread general biomarkers useful to evaluate the activation of the antioxidant system and the genotoxicity respectively, due to exposure to contaminants or mixture of them. The TOSC (Total Oxyradical Scavenging Capacity) Assay allows to quantify the capacity of the entire antioxidant system to neutralize the different forms of reactive oxygen species (ROS), which can cause damage to lipids, DNA and proteins if not kept under control.

Regarding the MN, their occurrence is an irreversible genotoxic event that reflects an accumulation of DNA breakages during cell cycle processes (Gorbi et al. 2009; Bolognesi and Fenech 2012). The micronucleus test is frequently used to evaluate the genotoxicity of contaminants due to its simplicity, reliability and sensitivity (Ayllon and Garcia-Vazquez 2000) in vertebrates (Çelik et al. 2005; Lajmanovich et al. 2005; Hui Yin et al. 2008; Hussain et al. 2012) and invertebrate species (Bolognesi and Fenech 2012), and rarely applied in reptiles (mainly turtles and crocodiles) (Poletta et al. 2009; Strunjak-Perovic et al. 2010; Poletta et al. 2011).

### Biomarkers assays

AChE activity was measured in supernatant (S10) brain cytosolic fractions, while total antioxidant capacity and GSTs activities were measured in S10 liver cytosolic fractions after homogenization (1:10, w/v) in 100 mM Tris–HCl buffer, 0.25 M sucrose and 1 mM EDTA (pH = 7.6) centrifuging at 10,000 g for 20 min at 4 °C (Amaral et al. 2012a). All the analyses were performed using the obtained supernatant preserved at −80 °C. AChE activity was determined spectrophotometrically in the brain following Ellman et al. (1961) slightly modified. The reaction medium was 50 mM Na-phosphate buffer (pH 7.4) and acetylthiocholine iodide (0.5 mM) as substrate. This latter is converted in thiocholine reacting with 5,5-dithio-bis-2-nitrobenzoic acid (0.2 mM DTNB). The activity was detected by recording the rate of absorbance at 412 nm and 18 °C during a 1-min period with a CARY50 spectrophotometer. The activity was normalized by total protein concentration (Lowry et al. 1951) and expressed as nmol/min/mg protein, using a molar extinction coefficient (ε) of 13.6 mM^−1^cm^−1^.

Glutathione S-transferase kinetic was determined spectrophotometrically (*λ* = 340 nm, at constant temperature 18 °C) with the method described in Habig et al. (1974), slightly modified by Regoli et al. (2012). The reaction medium was 100 mM K-phosphate buffer (pH = 6.5) with 1.5 mM reduced glutathione (GSH), 1.5 mM 1-chloro-2,4-dinitrobenzene (CDNB) as substrate and the cytosolic extract of the liver sample. The activities were normalized with total protein content (Lowry et al. 1951) and expressed as μmoli/min/mg protein.

TOSCA ROO^.^–TOSCA HO^.^ were detected according to Regoli and Winston (1999). The assay is based on the reaction between peroxyl and hydroxyl radicals artificially generated (at a constant rate) with the substrate α-keto-γ-methiolbutyric acid (KMBA) which is gradually oxidized to ethylene. The formation over time of ethylene was monitored by gas chromatography. The total antioxidant capacity (TOSC) of a sample against free radicals is quantified by its capability to inhibit ethylene formation compared to a control reaction. Indeed, the efficiency of cellular antioxidants as scavengers of the radicals produced is ground on their ability to reduce the reaction between free radicals and KMBA. Peroxyl radicals were generated by thermal homolysis of 20 mM 2-2’-azo-bis-(2methylpropionamidine)-dihydrochloride (ABAP) in 100 mM K-phosphate buffer (pH 7.4) and 0,2 mM KMBA. Hydroxyl radicals were produced through Fenton reaction at 35 °C of iron-EDTA (Fe^3+^ 0.18 µM / EDTA) and ascorbic acid. The final test conditions were: KMBA (0.2 mM), ascorbic acid (0.18 mM), Fe^3+^ (0.18 µM) and EDTA (0.36 µM) in K-phosphate buffer (100 mM) a pH 7.4. Both reactions were carried out in a final volume of 1 ml at 35 °C, in 10-ml vials sealed with gas-tight Mininert valves for multiple injections. Ethylene production was analysed by measuring 200 µl aliquots of headspace of the reaction vials at 15 min intervals with a gas chromatograph (HFC500) and a flame ionization detector (FID). The oven, injection, and FID temperatures were respectively, 35, 160, and 220 °C. Helium was the carrier gas. The TOSCA value of each sample was expressed in equivalents of GSH/gr of tissue, calculated using a calibration curve having as standard the TOSC values of three known GSH concentrations and normalized per gr of tissue.

Micronucleus (MN) frequencies were measured following Gorbi et al. (2009). Blood samples were exposed to fixative solution changes before setting up blood smears coloured with ethidium bromide (2 μg/ml). At least 1000 cells for each sample were examined under a fluorescence microscope to determine the percentage of cells containing MN on total cells.

### Parasite analyses

Even if less exploited, pesticides effects on parasite infections can be useful tools to evaluate the vulnerability of reptiles to environmental contaminants (Hopkins 2000; Marcogliese 2005). Organisms already affected by different environmental stresses seem to be more susceptible to infection by parasites (Marcogliese 2005). Changes in parasite load and composition represent a good bioindicator of environmental stress. The consequences of parasite infection and contamination have been deeply studied in birds (Mandal et al. 1986) and amphibians (Mann et al. 2009; King et al. 2010), but infrequently in reptiles (Amaral et al. 2012a; Soliman 2012).

Stomach, small and large intestine were rinsed with distilled water, opened and their contents examined for parasites utilizing a stereo microscope. Parasites were isolated and fixed in 96% ethanol. Helminths were identified to the lowest possible taxon (usually species). Parasite prevalence and mean abundance were calculated following Bush et al. (1997). Shannon’s diversity index was used to evaluate parasite community structure. This index refers to the relative frequency of each individual species detected in the sample area. Typical values in ecological studies are generally between 1.5 and 3.5, and the index is rarely greater than 4. The Shannon index increases as both the richness and the evenness of the community increase. Differences in parasites species richness and diversity among treatments were tested by rarefaction.

### Data analyses

To estimate the body condition (BC), the scaled mass index was calculated as described by Peig and Green (2009), since it represents an improvement over existing condition indices based on mass and length data (Peig and Green 2010). An analysis of variance (ANOVA) was used to detect differences between individuals from different treatment management, with BC as dependent variable, treatment (conventional, organic and control) and population ID (nested within treatment) as the independent variables. As the study species displays a sexual dimorphism affecting different body proportions (Schulte 2008), and females’ body condition could be influenced by their reproductive status, individuals were divided according to gender, and only males were analysed.

For each biomarker, the different responses obtained were examined using three-way mixed model ANCOVA designs with sex, treatment (conventional, organic and control), population ID (nested within treatment) as independent factors, BC as covariate, and the biomarker outputs as the response variables. All interaction effects were also included. When necessary, data were log-transformed to meet normality. Tukey’s post hoc test was applied to assess the significance of differences between pairs of group means.

Since in this species the body size differs between sexes with males being significantly larger than females, we analysed the difference of parasite load between sex and then we made a correlation between parasite load and SVL of females and males separately. Furthermore, based on infectious status, lizards were grouped into two categories, (uninfected/infected), to determine if the biomarkers could be affected by lizard infection status, different treatments types and/or their combinations (Marcogliese et al. 2009). We performed an ANOVA where infection status and treatment were considered as independent factors. Since each of the five considered biomarker were analysed separately, a sequential Bonferroni correction was used to account for multiple testing. Kruskal-Wallis test was applied to test differences of parasites prevalence and abundance.

Statistical analyses were performed by using Statistica (v8.0; StatSoft Inc 2007) and PAST v3.0 (Hammer et al. 2001) with alpha set at 0.05.

## Results

### Body condition

Overall, 80 adult lizards were captured. No significant differences in BC were observed across treatments (ANOVA, *F* = 1.08, *p* = 0.35) (Table 2 “Appendix”).

### Biomarkers analysis

Mean values (±standard deviation) of biochemical responses analysed in *P. siculus* tissues are shown in Table 2. No significant effect in inhibition of AChE, GSTs activity and in total antioxidant capacity towards peroxyl radicals (TOSCA ROO^.^) among treatment and populations or sex was observed (Table 3). TOSCA HO^.^ differed among treatments, with lizard from conventional field showing a higher oxidative stress by hydroxyl radicals than control and organic, and between sexes, with males presenting higher oxidative stress also by hydroxyl radicals than females (Table 3). T2 population showed the highest total antioxidant capacity (Post hoc Tukey’s test *p* < 0.05). As for MN test, lizards collected in conventionally treated fields showed a significant higher erythrocyte micronucleus frequency (especially in the T2 population) than lizards collected elsewhere (*p* < 0.05). No significant difference was found between organic and control farming fields (Post hoc Tukey’s test *p* ≥ 0.40) (Table 3)

**Table 2:** Mean values ± standard deviations of biomarker responses in *P. siculus* in conventional (T1 and T2), organic (B1 and B2) and control (C) sites

	Conventional		Organic		Control
Biomarker*s*	T1	T2	B1	B2	C
AChE (nmol/min/mg protein)	30.38 ± 12.72	34.80 ± 13.10	33.90 ± 12.36	32.51 ± 8.19	30.71 ± 10.15
GSTs (μmol/min/mg protein)	526.80 ± 160.29	517.20 ± 184.80	603.80 ± 222.42	618.30 ± 288.79	651.70 ± 215.90
TOSCA ROO^.^ (GSHeq/g tissue)	870.6 ± 247.0	824.8 ± 237.0	750.2 ± 142.7	932.9 ± 232.9	808.9 ± 286.8
TOSCA HO^.^ (GSHeq/g tissue)	1568.8 ± 365.3	2399.4 ± 386.0	1738.2 ± 261.6	1741.2 ± 360.9	1817.3 ± 461.7
MN (‰)	1.4 ± 0.4^a^	2.4 ± 0.3^a^	0.7 ± 0.3^a^	1.2 ± 0.3^a^	0.8 ± 0.2^a^

*AChE* acetylcholinesterase, *GSTs* glutathione S-transferases, *TOSCA* total oxyradical scavenging capacity assay towards peroxyl (ROO^.^) and hydroxyl (HO^.^) radicals, *MN* micronuclei frequency

^a^standard error

**Table 3:** Effects of treatment (conventional vs organic vs control), population (nested within treatment), sex, and all 2 * 2 interaction effects on biomarkers values in *P. siculus*

Biomarker	*df*	*F*	*p*
AChE			
Intercept	1	92.50	<0.0001
BC	1	0.60	0.442
Treatment	2	0.11	0.892
Population (treatment)	2	0.14	0.865
Sex	1	1.15	0.288
Treatment*sex	2	0.91	0.406
Population (treatment)*sex	2	2.61	0.081
Error	62		
GSTs			
Intercept	1	273.64	<0.0001
BC	1	1.55	0.217
Treatment	2	1.93	0.153
Population (treatment)	2	0.09	0.915
Sex	1	2.49	0.119
Treatment*sex	2	1.06	0.354
Population (treatment)*sex	2	0.86	0.428
Error	64		
TOSCA ROO			
Intercept	1	19.35	<0.0001
BC	1	0.67	0.415
Treatment	2	0.44	0.648
Population (treatment)	2	1.68	0.196
Sex	1	1.22	0.273
Treatment*sex	2	0.22	0.788
Population (treatment)*sex	2	0.06	0.942
Error	61		
TOSCA HO^.^			
Intercept	1	21.70	<0.0001
BC	1	2.44	0.128
Treatment	2	4.66	**0.025**
Population (treatment)	2	1.28	0.305
Sex	1	14.28	**0.002**
Treatment*sex	2	0.25	0.780
Population (treatment)*sex	2	0.76	0.482
Error	16		
MN			
Intercept	1	4.42	<0.0001
BC	1	0.56	0.455
Treatment	2	9.71	**<0.0001**
Population (treatment)	2	2.14	0.127
Sex	1	1.20	0.277
Treatment*sex	2	0.07	0.931
Population(treatment)*sex	2	0.86	0.430
Error	62		

Significant values are reported in bold

### Parasite analyses

Overall, four helminth parasite species were identified: the nematodes *Spauligodon sp*. Skrajabin, Schikhobalova, and Lagodovskaja 1960 and *Skrjabinelazia* sp. Sypliaxon 1930, the cestodes *Nematotaenia tarentolae* Lopez-Neyra 1944, and larval forms of *Mesocestoides litteratus* (Batsch 1786). The total number of identified species per lizard varied between 1 and 3, with prevalence values ranging from 2.9 to 57%, while mean abundance was 0.03–1.76 worms per individual (Table 4). Only *Spauligodon* sp. was found in all the treatments and it was numerically dominant representing 100, 98 and 50 % of all parasites found in organisms from conventional, organic and control areas, respectively. Parasite’s prevalence and mean abundance did not differ significantly when treatments were compared (prevalence, Kruskal–Wallis *H*_2,5_ = 1.40 *p* = 0.50; mean abundance, *H*_2,5_ = 0.60 *p* = 0.74). Females showed a higher parasite load than males (Mann–Whitney *U* Test, *U* = 590, *p* = 0.04). However no correlation between SVL and parasite load was found in each treatment and in their total both in females (*R*_Spearman_ ≤ 0.06, p ≥ 0.22) and males (*R*_Spearman_ ≤ 0.18, *p* ≥ 0). No effect of infection level (alone or as interaction with treatments) on the biomarkers analysed in the study was observed. Only MN responded to treatments alone besides of infection status with a higher presence of MN in conventional fields (Post hoc Tukey’s test, *p* < 0.001) (Table 3 “Appendix”).

**Table 4:** Community composition, prevalence (P%) and mean abundance (MA) (SE in parenthesis) of helminths observed in *P. siculus* in conventional, organic and control sites

	Conventional		Organic		Control	
No. of species	1		3		2	
Shannon–Weiner index	0		0.6		0.69	
Helminth species	P%	MA	P%	MA	P%	MA
Nematodes						
* Spaulingodon* sp.	31	1.76 (0.99)	57	3.11 (1.22)	13	0.31 (0.25)
* Skrjabinelazia* sp.	–	–	–	–	19	0.31 (0.25)
Cestodes						
* Nematotenia tarentola*	–	–	2.9	0.03 (0.02)	–	–
* Mesocestoides litteratus*	–	–	2.9	0.03 (0.02)	–	–

Number (No) of parasite species and Shannon–Wiener diversity index are also shown

## Discussion

This study represents a preliminary investigation on health of lacertid lizard exposed to a mix of pesticides in agricultural environments in Central Italy.

Body condition is a morphological stress index, providing useful information about physiological status and general health status of an organism (Stevenson and Woods 2006). Indeed, the decrease of body condition can have important consequences for an individual’s fitness, including capacity to survive hibernation, ability to compete for breeding opportunities, fecundity, and capacity to fight disease (Amo et al. 2006, 2007; Pafilis et al. 2009). However, no clear demonstration of a direct link between toxic effects from pesticide exposure and body condition in reptiles has been provided yet. To our knowledge, only few studies reported a decrease in body condition index in lizards *P. muralis* and *P. bocagei* inhabiting agricultural areas exposed to pesticides (Amaral et al. 2012b; Mingo et al. 2017). Further analyses will be necessary to better disentangle these issues.

Regarding the biomarker analysis, no inhibition of AChE activity and alteration of GSTs were detected in lizards after pesticide application, when compared among treatments and populations. This implies that (i) the pesticides involved in this study may not be neurotoxic and may not be detoxified by GSTs activities; (ii) the studied lizards may have developed a fast enzyme recovery activity from intoxication (Mingo et al. 2016) or a tolerance capacity toward those contaminants to which they have been chronically exposed; (iii) the pesticides concentrations were not high-enough to cause an effect on the measured endpoints; (iv) sampling events took place too late compared to pesticides application date in order to detect fast molecular biomarkers reactions (i.e., GST, AChE) (Mingo et al. 2016). Further studies therefore are needed to clarify if there is a neurotoxicity involving other esterase (i.e. butyryl cholinesterase), and which pathway is involved in pesticides detoxification processes (either using a different kind of biomarkers such as those of phase I metabolizing enzyme system such as cytochrome p450 enzymes activities) through exposure studies with different pesticides concentrations and under controlled condition.

Oxidative stress, evaluated in terms of total antioxidant capacity (TOSC) against specific free radicals, was induced in the study lizards. A significant response of TOSCA HO^.^ values was detected among treatments, especially in one conventional field (i.e., T2). This suggests that lizards considered in this study have promptly activated the antioxidant system to counteract ROS formation due to synthetic pesticide exposure and that the oxidative stress should be ascribed to hydroxyl radicals rather than to peroxyl ones. In order to better clarify this mechanism of action, individual components of the antioxidant system, such as catalase (CAT) and glutathione peroxidase (GPx) activities, could be analysed. Indeed, these enzymes are both involved in detoxification of hydrogen peroxide which is an important precursor to hydroxyl radicals (Di Giulio et al. 1995). Concerning possible intersexual differences as response to pesticide exposure, we found higher antioxidant levels in males than in females, as already observed in the *Crocodylus niloticus*, Laurenti 1768 (Arukwe et al. 2015). Sexes can be subject to distinctive chemical effects because of different pathways of exposure, uptake, and metabolism. Moreover, irrespectively of the presence of xenobiotic substances, also stresses arising from predation risk and territoriality are known to induce oxidative stress (Pinya et al. 2016). In *P. siculus*, males are bigger and heavier than females and are characterized by aggressive territorial behaviour (Corti et al. 2011). This may suggest that males are more likely exposed to different contaminants or more prone to increased oxidative stress than females, because of their higher activity levels in searching for food and patrolling their territory. However, how the effects of chemicals could be altered by gender susceptibility is still a novel research field with available data on few reptiles species (i.e., *Alligator mississippiensis* (Daudin, 1801); *Trachemys scripta* (Thunberg in Schoepff, 1792)) (Burger et al. 2007).

The production of free radicals in exposed lizards has probably exceeded the defence of the antioxidant system leading to DNA damages. These damages are underlined by a higher micronuclei frequency, recorded in red blood cells of lizards from conventional areas compared to the remaining fields, especially in the T2 field where the thiophanate methyl was also used. These results agree with Capriglione et al. (2011) documenting the occurrence of genotoxic effects in *P. siculus* exposed to thiophanate methyl fungicide. The genotoxicity is particularly relevant because of the delayed appearance (months or years) of fully manifested genotoxic effects (i.e. malignant tumours, decreased reproductive success and altered genotypic diversity) which may be significant at population and community levels (Gardner and Oberdorster 2016). Differences in responses of animals by the two conventional areas may be due to the different pesticide administration: while lizards in T2 field were exposed to four different chemicals, just two were applied in T1 with lower concentration dose than T2 (Table 1). Thus, one possible explanation could be that treatment with tebuconazole and lambda-cyhalothrin, as in T1, does not induce measurable toxicological outcomes in our lizards; instead, treatment with tebuconazole together with lambda-cyhalothrin, thiophanate-methyl and deltamethrin, as in T2, at higher concentration than T1, induces measurable toxicity. Since no differences were found between control and organic farming areas, we could hypothesize that the exposure to copper sulphate, in the amount and frequency applied in the organic farms here investigated does not induce toxicity in lizards analysed in this study. In order to potentially detect copper/heavy metals in organs and tissues as long-term effects, further studies may investigate copper bioaccumulation rate by spectrometric analyses (e.g. GC/MS, LC/MS or AAS) (Oyekunle et al. 2012; Sparling 2016).

Several studies have documented how some pesticides (or a mixture of pesticides) can compromise immune systems inhibiting critical enzymes or damaging immune organs of invertebrates (i.e., earthworms *Eisenia andrei*) and vertebrates (i.e., bird *Phasianus colchicus* Linnaeus, 1758) (Galloway and Handy 2003), which become more vulnerable to infections from different parasitic form as trematodes (Kiesecker 2002; Linzey et al. 2003) and nematodes (Christin et al. 2003, 2004; Gendron et al. 2003). Our results are consistent with the general low rates of parasite intestinal infection observed in lacertid lizards, *P. bocagei* and *P. carbonelli*, Pérez-Mellado 1981 (Roca et al. 2006; Roca and Galdón 2010). Specifically, two nematode species with a direct life cycles (Casanova et al. 2003; Lhermitte et al. 2008; Yildirimhan et al. 2011) were found in conventional fields, while two cestode species with a complex life cycles (Roca and Hornero 1994; Sargsyan et al. 2014) were detected in control and organic fields. Similarly, a study conducted on an amphibian (the American bullfrog, *Lithobates catesbeianus* (Shaw 1802)) showed a lower species richness, a higher abundance of direct life cycle nematodes and a lower diversity of parasite requiring multiple-host life cycle in pesticide polluted wetlands (King et al. 2010). It is likely that in the conventional areas, pesticides affected in some way cestodes (at different life stages) and/or their hosts (i.e., mammals, birds, or lizards), thus decreasing the success and reproduction of multiple-host parasites. However, we could not detect a clear association between pesticides application and intestinal parasite abundance. Studies on leopard frogs (*Rana pipiens* (Schreber 1782)) (Christin et al. 2003; Gendron et al. 2003), revealed how nematode parasites (i.e., *Rhabdias ranae*) may alter the expression of biomarkers (i.e., lymphocyte proliferation) that are routinely used to evaluate individual animal health in environmental studies, thus possibly affecting their interpretations for studies in toxicology and conservation biology. By contrast, our study does not provide evidence of relationship between biomarkers and lizard infectious status, so that such confounding effects could be reasonably discarded. Noteworthy, in this study, *P. siculus* was recorded as a new host for the larval forms of *Mesocestoides litteratus* (Batsch 1786) (Berrilli and Simbula 2020). The complete life cycle of the genus *Mesocestoides* Vaillant 1863, although it is not still clear, probably requires two intermediate hosts and a definitive one. In the second intermediate hosts (commonly reptiles, amphibians, birds, and micro-mammals), the tetrathyridium stage occurs (Conn 2004; Conn and Świderski 2013) and in some cases may asexually reproduce by spreading within the peritoneal cavity and provoking severe systemic infections. Until now, tetrathyridia forms of *Mesocestoides* were sporadically observed in Italy, infecting domestic cats (Jabbar et al. 2012), dogs (Bonfanti et al. 2004), and a callitrichid monkey (Montalbano Di Filippo et al. 2018). The severe infection here observed indicates the circulation of *M. litteratus* in this country, with a potential risk of infection for wild and domestic animals. Further extensive samplings on intermediate and definitive hosts will be needed to confirm the presence and the distribution of this parasite in the region.

In conclusion, our experimental study, combining different approaches, highlighted that some synthetic pesticides (i.e. tebuconazole, lambda-cyhalothrin, thiophanate-methyl, deltamethrin) may induce toxic effects in *P. siculus* if used at high concentration in the same field. Moreover, it revealed a pesticides-mixture induction of hydroxyl radicals in lizards, that was not completely counteracted by defence mechanisms (i.e. antioxidant system), translating into a distinctly negative effect on lizard’s DNA, with potential damaging outcomes in the long-term. Given that lizards in agricultures could be exposed to pesticides with different modalities, we suggest to perform additional field and laboratory studies on *P. siculus*, testing one or more pesticides at different concentrations and analysing other biomarkers, to confirm *P. siculus* as a good model species in ecotoxicological studies, to achieve a more accurate evaluation of specific pesticide effects, and to clarify which defence mechanism could be activated in relation to well-known exposure history.

## Supplementary information

Supplementary Materials
