# Supplementary material for: Biological responses in pesticide exposed lizards (Podarcis siculus)
Source: Ecotoxicology. 2021 Jun 26;30(6):1017–28. doi: 10.1007/s10646-021-02440-3 (PMC8295152; doi:10.1007/s10646-021-02440-3)
Supplement: Supplementary file 1 — Supplementary Materials [file 10646_2021_2440_MOESM1_ESM.docx]

**Table 1.** Data on pesticide formulations application dates and lizard sampling dates in all the studied sites during the year 2018.

| Treatment | Area | Chemical | Application date | Sampling date |
| --- | --- | --- | --- | --- |
| Conventional | T1 | Ares 430sc® | 28/05 - 26/06 | 03/07 |
|  |  | Sparviero® | 28/05 - 26/06 |  |
|  | T2 | Ares 430sc® | 31/05 | 10/07 |
|  |  | Copper 40% | 31/05 |  |
|  |  | Sparviero® | 31/05 – 03/07 |  |
|  |  | Enovit® | 17/06 – 03/07 |  |
|  |  | Glorial 25ec® | 17/06 |  |
| Organic | B1 | Copper | 19/06 | 26/06 |
|  | B2 | Copper | 20/07 | 27/07 |
| Control | C | - | - | 24/07 |

| Treatment | Area | M-F | SVL (mm) | | | BM (g) | | BC(g/mm) | | | |
| --- | --- | --- | --- | --- | --- | --- | --- | --- | --- | --- | --- |
|  |  |  | M | F | M | | F | | M | F |  |
| Conventional | T1 | 3-10 | 75.7±0.29 | 73.2±0.55 | 7.36±0.53 | | 5.96±0.90 | | 9.08±1.18 | 5.83±0.39 |  |
|  | T2 | 7-9 | 84.0±0.49 | 70.7±0.44 | 9.91±1.67 | | 5.61±0.91 | | 9.23±0.85 | 5.94±0.56 |  |
| Organic | B1 | 11-9 | 78.2±0.78 | 70.1±0.41 | 8.11±1.89 | | 5.79±1.15 | | 9.08±1.06 | 6.09±0.67 |  |
|  | B2 | 9-6 | 83.2±0.46 | 73.9±0.44 | 9.06±0.95 | | 5.45±0.83 | | 8.67±1.42 | 5.18±0.43 |  |
| Control | C | 9-7 | 84.7±0.29 | 75.1±0.49 | 10.0±0.80 | | 5.42±0.72 | | 9.18±0.97 | 5.00±0.41 |  |

**Table 2.** Sample size (n) for males (M) and females (F) for all populations studied**.** Mean values ± standard deviations of snout-vent length (SVL), body mass (BM) and body condition (BC).

**Table 3**. Effects of treatment and parasite infection on 5 biomarkers of *P. siculus* health. Significant values are reported in bold; Asterisks (*) indicate a significant effect survived to Bonferroni correction (p <0.01).

| Biomarkers | Treatment | Parasites | Treatment*Parasites |
| --- | --- | --- | --- |
| AChE | F=0.11; p=0.89 | F=0.46; p=0.50 | F=0.31; p=0.73 |
| GSTs | F=0.40; p= 0.67 | F=6.99; p=0.14 | F=0.55; p=0.58 |
| TOSCA ROO^.^ | F=0.30; p=0.74 | F=2.42; p=0.12 | F=0.10; p= 0.90 |
| TOSCA HO^.^ | F=0.25; p= 0.78 | F=1.78; p= 0.20 | F=0.65; p=0.53 |
| MN | F=7.83; **p<0.001*** | F=0.29; p=0.59 | F=0.51; p=0.60 |
